# Adoptive Transfer of Photosensitizer-Loaded Cytotoxic T Cells for Combinational Photodynamic Therapy and Cancer Immuno-Therapy

**DOI:** 10.3390/pharmaceutics15041295

**Published:** 2023-04-20

**Authors:** André-René Blaudszun, Woo Jun Kim, Wooram Um, Hong Yeol Yoon, Man Kyu Shim, Kwangmeyung Kim

**Affiliations:** 1 Medicinal Materials Research Center, Biomedical Research Division, Korea Institute of Science and Technology (KIST), Seoul 02792, Republic of Korea; 2 Biosensor and Materials Group, KIST Europe Forschungsgesellschaft mbH (KIST Europe), Saarland University, 66123 Saarbrücken, Germany; 3 College of Pharmacy, Graduate School of Pharmaceutical Sciences, Ewha Womans University, Seoul 03760, Republic of Korea

**Keywords:** cancer immunotherapy, photodynamic therapy, combination therapy, adoptive T cell therapy, cell-mediated drug delivery

## Abstract

Adoptive cell transfer (ACT) has shown remarkable therapeutic efficacy against blood cancers such as leukemia and lymphomas, but its effect is still limited due to the lack of well-defined antigens expressed by aberrant cells within tumors, the insufficient trafficking of administered T cells to the tumor sites, as well as immunosuppression induced by the tumor microenvironment (TME). In this study, we propose the adoptive transfer of photosensitizer (PS)-loaded cytotoxic T cells for a combinational photodynamic and cancer immunotherapy. Temoporfin (Foscan^®^), a clinically applicable porphyrin derivative, was loaded into OT-1 cells (PS-OT-1 cells). The PS-OT-1 cells efficiently produced a large amount of reactive oxygen species (ROS) under visible light irradiation in a culture; importantly, the combinational photodynamic therapy (PDT) and ACT with PS-OT-1 cells induced significant cytotoxicity compared to ACT alone with unloaded OT-1 cells. In murine lymphoma models, intravenously injected PS-OT-1 cells significantly inhibited tumor growth compared to unloaded OT-1 cells when the tumor tissues were locally irradiated with visible light. Collectively, this study suggests that combinational PDT and ACT mediated by PS-OT-1 cells provides a new approach for effective cancer immunotherapy.

## 1. Introduction

Cancer immunotherapy that redirects and boosts the natural functions of the immune system to fight a variety of malignant neoplasms has progressively changed the paradigm of cancer treatment. In addition to the classical modalities, such as surgery, radiation therapy and chemotherapy, immuno-oncology is now considered to be a promising approach for the complete remission of cancer. One of the effective cell-mediated immunotherapies is adoptive cell transfer (ACT), which predominantly uses patient’s own T lymphocytes that are genetically engineered to express either the well-defined T cell receptors (TCRs) [1,2] or chimeric antigen receptors (CARs) specific to tumor-associated antigens [3,4,5]. In principal, ACT includes the isolation of autologous T cells, followed by their ex vivo activation, as well as genetic transformation, expansion and, finally, re-infusion into the patient’s blood stream [6,7]. Ideally, redirected effector T cells selectively recognize and eradicate malignant tissues without affecting healthy cells. The effector T cells promote the apoptosis of tumor cells through two principal contact-dependent cytolytic mechanisms [8]. The first one acts through tumor-cell-penetrating effector molecules through the synergy of the pore-forming protein perforin and proteases. The second one requires the binding of trimeric Fas (CD95) on the target tumor cells with FasL (CD95L) expressed on T cells [9]. In addition, the memory cells generated during ACT induce long-lasting antitumor immunity to prevent the relapse and metastasis of malignancies. Indeed, in several clinical trials, the lives of many patients with late-stage cancers were prolonged, indicating the curative potential of adoptive therapy using genetically engineered T cells [10].

ACT has shown remarkable therapeutic efficacy against blood cancers such as leukemia and lymphomas in clinic, but its effectiveness is still limited due to the lack of well-defined antigens expressed by aberrant cells within tumors, the insufficient trafficking of the administered T cells to the tumor sites, as well as the immunosuppression induced by the tumor microenvironment (TME) of solid cancers [11,12]. An alternative approach to improving the efficacy of ACT is the use of T lymphocytes as living drug-delivery vehicles [13]. In several other studies, researchers have evaluated the feasibility of enhancing the T cell killing capacity by loading cytotoxic compounds, such as chemotherapeutic drugs, into these cells [14,15,16]. The adoptive transfer of T cells loaded with ricin or 4′-deoxy-4′-iododoxorubicin (IDX) showed a superior therapeutic efficacy in advanced and metastatic tumors than unloaded T cells, owing to the combination of the T cells’ lytic activity with the efficient release of the loaded drugs. As these types of payloads seriously harm, and eventually kill, carrier T cells, there have been several attempts to temporarily protect T cells by entrapping the loading drugs into polymeric, as well as gold-shell-coated polymeric, nanoparticles; however, the uptake of those nanoparticles did not prevent the premature death of the T lymphocytes [17].

**Figure 1:**
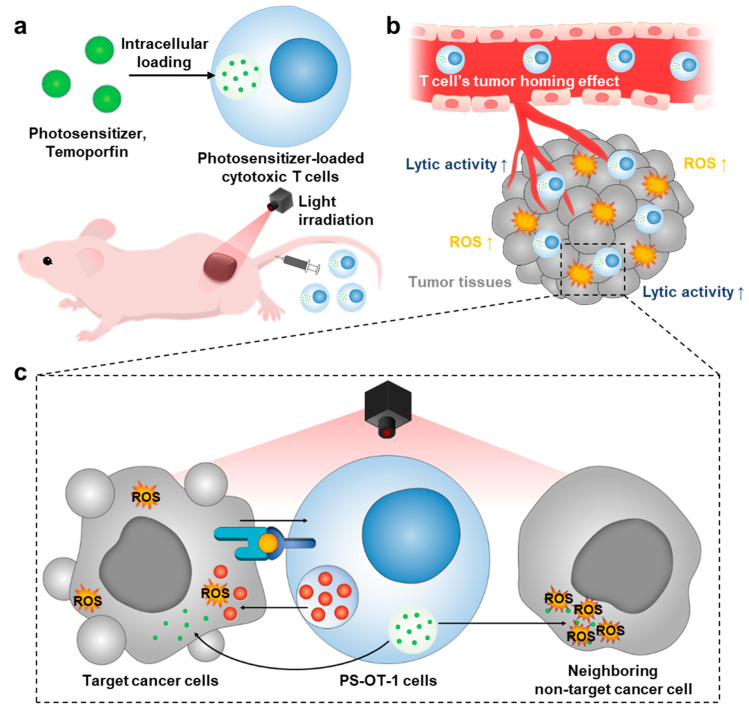
Adoptive transfer of photosensitizer (PS)-loaded cytotoxic T cells for combinational photodynamic therapy (PDT) and cancer immunotherapy. (**a**) Photosensitizer (PS) is loaded into cytotoxic T cells (OT-1 cells), resulting in PS-OT-1 cells. (**b**) When PS-OT-1 cells are intravenously injected into the tail vein, they accumulate within the targeted tumor tissues owing to T cells’ tumor homing effect. (**c**) Upon light irradiation, PS-OT-1 cells can effectively eradicate the tumor cells that are directly attacked by T cells, as well as neighboring non-target cancer cells that are not immediately recognized by T cells in the tumor tissues by combinational effect of PDT and ACT.

Herein, we propose the adoptive transfer of photosensitizer (PS)-loaded cytotoxic T cells (CTLs) for combinational photodynamic therapy (PDT) and cancer immunotherapy. The stimulus-sensitive PS can remain in a non-toxic state to prevent the occurrence of premature damage to the T cells, unless its cytotoxic activity is triggered by light irradiation. Therefore, T lymphocytes that deliver PS as living vehicles to targeted tumor tissues could be a promising therapeutic approach for combinational PDT and cancer immunotherapy [18]. The concept aims at combining the phototoxicity of cellular transported PS with the lytic activity of CTLs. First, the clinically applicable photosensitizer, Temoporfin (Foscan^®^), is loaded into murine cytotoxic T cells (OT-1 cells) via an intracellular loading method to achieve photosensitizer-loaded OT-1 cells (PS-OT-1 cells) (Figure 1a). Second, when the PS-OT-1 cells are intravenously injected, they accumulate within the targeted tumor tissues, owing to the T cells’ tumor homing effect (Figure 1b). Third, upon light irradiation, this strategy can effectively eradicate the tumor cells that are directly attacked by T cells, as well as the neighboring cancer cells in the tumor tissues, through the combinational effect of PDT and ACT (Figure 1c). To this end, OT-I cells served as model CTLs, and tumors grown from cells of the murine lymphoma cell line EG.7-OVA served as the targets. OT-I cells express a transgenic T-cell receptor (TCR), which recognizes chicken ovalbumin (OVA) peptide residues 257–264 (SIINFEKL) in the context of murine MHC class I molecule H-2K^b^ presented by E.G7-OVA cells [19]. Next, temoporfin, a porphyrin derivative that exhibits a prominent absorption peak at around 652 nm, is loaded into the OT-1 cells for a potent PDT efficacy [20]. The PS is efficiently loaded into the OT-1 cells via an intracellular loading method by simply incubating the PS with the cells in a culture. In this study, the methodology to prepare temoporfin-loaded OT-1 cells (PS-OT-1 cells) and their cytotoxic function under light irradiation are evaluated in vitro. In addition, the combinational effect of photodynamic and cancer immunotherapy on PS-OT-1 cells under local light irradiation is assessed in EG.7-OVA tumor-bearing mice.

## 2. Materials and Methods

### 2.1. Reagents

Temoporfin (Foscan^®^, 5,10,15,20-Tetrakis(3-hydroxyphenyl)chlorin (mTHPC)) was purchased from Cayman Chemical (Ann Arbor, MI, USA). Rat anti-mouse CD8 antibody was purchased from BD Bioscience (San Jose, CA, USA). Recombinant interleukin-2 (IL-2) was purchased from PeproTech (Cranbury, NJ, USA). RPMI 1640 medium, fetal bovine serum (FBS), penicillin and streptomycin were purchased from Welgene Inc. (Gyeongsangbuk-do, Republic of Korea). TUNEL assays kit was purchased from R&D systems (Minneapolis, MN, USA). Mouse T cell activation and expansion kit was purchased from Miltenyi Biotec (Bergisch Gladbach, Germany). Mouse CD8 T cell enrichment columns were purchased from R&D Systems, Inc. (Minneapolis, MN, USA). Cell counting kit-8 (CCK-8) and WST-1 colorimetric assays were purchased from Vitascientific (Beltsville, MD, USA) and Roche Diagnostics GmbH (Mannheim, Germany), respectively.

### 2.2. Preparation of OT-I Cells

Mice were bred under pathogen-free conditions at the Korea Institute of Science and Technology (KIST). All experiments with live animals were performed in compliance with the relevant laws and institutional guidelines of the Institutional Animal Care and Use Committee (IACUC) in KIST, and the IACUC approved the experiment (approval number of 2020-123). Five-week-old OT-I mice (Nara Biotech Co., Ltd., Seoul, Republic of Korea) were euthanatized, and their spleens and inguinal lymph nodes were collected. Then, secondary lymphoid tissues were homogenized under sterile conditions by grinding the organs through a cell strainer (40 µm, Falcon, Corning Inc., Oneonta, NY, USA). The obtained cell suspension was centrifuged at 300× *g* for 10 min, and the sediment was resuspended in 5 mL of lysis buffer (BioLegend, San Diego, CA, USA) to deplete red blood cells (RBC). The cell suspension was filtered through a cell strainer to remove RBC debris clots. CD8^+^ T cells were separated from other cells using the CD8^+^ T-cell enrichment column according to the manufacturer’s instructions. Briefly, approximately 1.8 × 10^8^ cells were resuspended in 2 mL of the provided column buffer (CB), and 1 mL of a monoclonal antibody cocktail was added to the cell suspension. For the negative selection of CD8^+^ T cells, the cell suspension was passed through a T Cell Subset Column. Finally, the cells were resuspended in RPMI 1640 cell culture medium supplemented with 10% (*v*/*v*) of FBS and 1% (*v*/*v*) of penicillin-streptomycin solution.

Next, the CD8^+^ T cells were activated using MACSiBeads conjugated with antibodies against murine CD3 and CD28 (MACS T Cell Activation/Expansion Kit, Miltenyi Biotec Inc., Auburn, CA, USA) according to the manufacturer’s instructions. In brief, activation beads were added to a suspension of OT-I cells at a bead-to-cell ratio of 1:1. In addition, the cell culture medium was supplemented with 30 U/mL of recombinant murine Interleukin-2 (IL-2, PeproTech Inc., Seoul, Republic of Korea), as well as 55 mM of 2-Mercaptoethanol (2-ME, Gibco, Life Technologies, Thermo Fisher Scientific Korea Ltd., Seoul, Republic of Korea). Finally, the beads were removed from the cultures through density gradient centrifugation using Ficoll-Paque Premium (Density 1.084 g/mL, GE Healthcare Bio-Sciences AB, Uppsala, Sweden). The purity of the expanded CD8^+^ T lymphocytes was determined through flow cytometry (BD Accuri C6, BD Biosciences, San Jose, CA, USA) using 2.5 µg/mL APC-conjugated antibodies against murine CD8 (BioLegend, Inc., San Diego, CA, USA) (Figure S1).

### 2.3. Preparation of Temoporfin (PS)-Loaded OT-1 Cells (PS-OT-1 Cells)

The optimal loading concentration of temoporfin was confirmed by measuring the cell viability of the OT-1 cells after treatment with different concentrations of temoporfin (12.5–100 µg/mL). After 2 h of incubation, the cell viability of the OT-1 cells was confirmed via cell counting kit-8 (CCK-8) assays. The cells were incubated with a culture medium containing 10% CCK-8 solution for 20 min, followed by analysis using a microplate reader and the measurement of the absorption at a wavelength of 450 nm. To load the temoporfin (Foscan^®^) into the OT-1 cells, the cells were incubated with 25 µg/mL of temoporfin in a cell culture medium at 37 °C for 2 h. Then, the PS-loaded OT-1 cells (PS-OT-1 cells) were sedimented at 300× *g* for 10 min to remove the non-loaded remaining temoporfin. The successful loading of temoporfin was confirmed by measuring the fluorescence intensities of the PS-OT-1 cells via a Guava easyCyte flow cytometer (Merck KGaA, Darmstadt, Germany) controlled by Guava^®^ Soft^TM^ 3.3 (Merck KGaA). The geometric means (GeoMean) of the fluorescence intensities (GMFI) derived from gated populations were calculated with FlowJo software (Tree Star, Ashland, OR, USA). The control values were subtracted from the corresponding sample values and then normalized.

To visualize the temoporfin uptake via confocal laser scanning microscopy (CLSM), 5 × 10^6^ PS-OT-1 cells were resuspended in flow cytometer buffer (PBS supplemented with 5% (*v*/*v*) FBS and 0.1% (*w*/*v*) NaN_3_ (Biosesang Inc., Incheon, Republic of Korea)). The cell membranes were stained by adding 25 µL of Alexa Fluor 488-conjugated antibodies against murine CD8α (R&D Systems, Inc., Minneapolis, MN, USA). After 30 min of incubation, the cells were fixed in 2 mL of PBS containing 4% (*v*/*v*) formaldehyde, as well as 0.1% (*v*/*v*) glutaraldehyde. Then, the nuclei of the cells were stained with DAPI (0.01 µg/mL in PBS, Invitrogen, Thermo Fisher Scientific Korea Ltd., Seoul, Republic of Korea) at room temperature for 5 min. Finally, fluorescence images were captured using a Leica TCS SP8 CLSM (Leica Microsystems GmbH, Wetzlar, Germany). To measure the temoporfin release from the PS-OT-1 cells, 1,3-diphenylisobenzofuran (DPBF) was used to measure the extracellular ROS generation of the temoporfin released from the PS-OT-1 cells over the incubation time. Firstly, freshly prepared PS-OT-1 cells (5 × 10^6^) were incubated in cell culture media at 37 °C for 2 days. Second, the cells at days 0, 1 and 2 were washed with fresh media 2 times. The washed PS-OT-1 cells were further incubated in fresh cell culture media at 37 °C for 6 h and the cell media samples were isolated by centrifuging the PS-OT-1 cells (300× *g*) for 10 min. Next, each cell media sample was dispersed in DMSO (1 mL) containing DPBF (10 μM) and histidine (1.2 mM). Then, each sample (*n* = 5) was irradiated for 1 min with a laser at a wavelength of λ = 655 nm (MRL-III-655-200mW, Changchun New Industries Optoelectronics Technology Co., Ltd., Changchun, China), wherein the samples were irradiated homogeneously with a power of 40 mW. Finally, the absorbance of DPBF was measured at 450 nm with a UV-Vis spectrometer (Agilent Cary 60, Agilent Technologies, Santa Clara, CA, USA).

### 2.4. Antitumor Efficacy of PS-OT-1 Cells In Vitro

The target cells (EL-4/E.G7-OVA) were seeded into 96-well cell culture plates (round bottom, Corning Inc., Corning, NY, USA) and subsequently treated in triplicate, either with effector OT-I cells or PS-OT-I cells, at an effector to target cell ratio of 2:1 (80,000 to 40,000 cells). As the negative control, cancer cells were incubated only with a cell culture medium. Corresponding background controls were also prepared. Two hours before the absorbance measurements, co-cultures were irradiated with a fluence of 8.3 J/cm^2^ applied over a period of 15 min using a tungsten halogen light source (Haloline Eco, 400 W, 9000 lm, Osram, München, Germany). WST-1 assays were started 30 min after illumination. Then, the cells were incubated for a further 75 min with 10 µL of WST-1 reagent. All co-cultivation periods, of 8 h or 24 h in total, were conducted at 37 °C in a humidified atmosphere containing 5% CO_2_ and protected from light. Before measuring the absorbance (A) at a wavelength of 450 nm, the samples were shaken. The values of the mean absorbance derived from the negative control samples represented 100% bio-reductive activity (viability). The percentage of cancer cell viability was calculated via the following equation:

$$\%\;\mathrm{viability}=100\;\times \;\frac{\left(A\;\mathrm{of}\;\mathrm{co-cultures}\right)-\;(A\;\mathrm{of}\;\mathrm{effectors}\;\mathrm{alone})}{\left(A\;\mathrm{of}\;\mathrm{targets}\;\mathrm{alone}\right)}$$

### 2.5. Antitumor Efficacy of PS-OT-1 Cells in a Murinelymphoma Model

To prepare lymphoma tumors, 4 × 10^6^ EG.7-OVA cells were subcutaneously injected into BALB/C nude mice (*n* = 5, 5 weeks old, Nara Biotech Co., Ltd., Seoul, Republic of Korea). Before the treatment of the animals, the overall tumor volume was measured and the mice were accordingly divided into groups to reduce the differences in the tumor burden between groups. When the tumor volumes reached a mean volume of around 100 mm^3^ per group, the mice were treated with 5 × 10^6^ unloaded or PS-loaded OT-1 cells. Then, 6 h after injection, all tumors were irradiated with 200 J/cm^2^ applied over a period of 10 min via a laser (MRL-III-655-200mW, Changchun New Industries Optoelectronics Technology Co., Ltd., Changchun, China) emitting light with a wavelength of 655 nm. The tumor volumes were monitored and determined daily using a caliper. The greatest longitudinal diameter (*l*) and the greatest transverse diameter (*w*) were measured. The tumor volumes (V) were calculated by the following modified formula for an ellipsoid: V = 0.52 × *l* × *w*^2^. The mice were euthanized when the tumor volumes exceeded approximately 2000 mm^3^.

### 2.6. Statistical Analysis

Statistical significance between the experimental and control groups was analyzed using Student’s *t*-test. One-way analysis of variance (ANOVA) was used for comparisons of more than two groups, and multiple comparisons were performed using a Tukey-Kramer post-hoc test. All results are expressed as means ± SD (standard deviation), and *p* values < 0.05 were considered statistically significant.

### 2.7. Data Availability

All of the relevant data are included in this published article and its Supplementary Information files. The datasets generated during and/or analyzed during the current study are available from the corresponding author on reasonable request.

## 3. Results and Discussion

### 3.1. Preparation of Temoporfin-Loaded OT-1 Cells (PS-OT-1 Cells)

**Figure 2:**
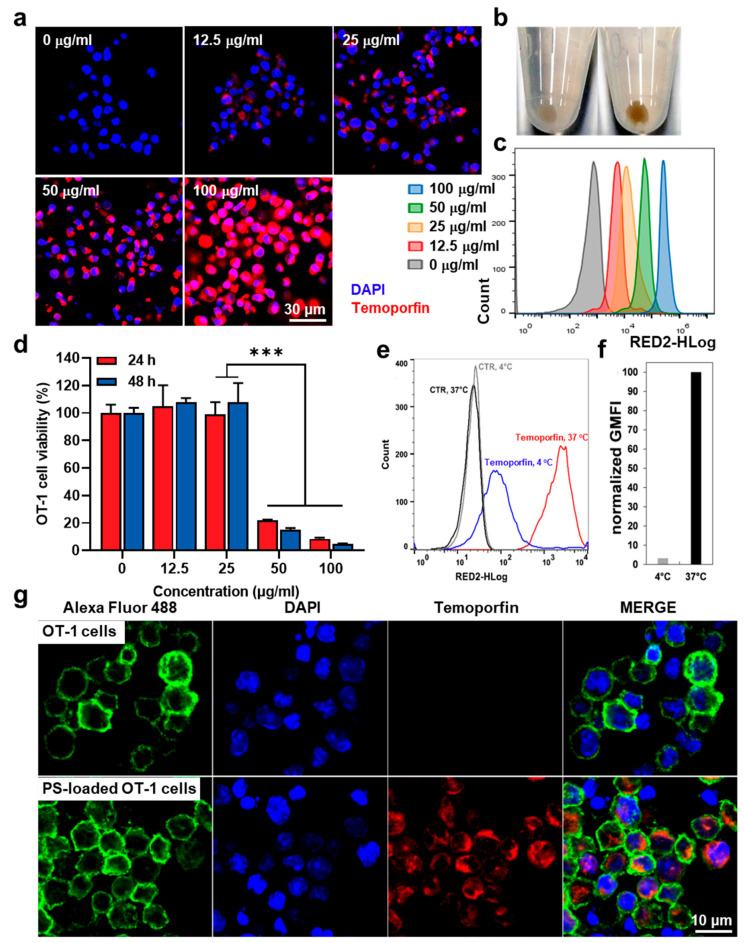
Preparation of temoporfin-loaded OT-1 cells (PS-OT-1 cells). (**a**) Fluorescence images of OT-1 cells after incubation with different concentrations of temoporfin from 12.5–100 μg/mL. (**b**) Cell sediment of PS-OT-1 cells or unloaded OT-1 cells that are incubated with 25 μg/mL for 2 h or unloaded OT-1 cells. (**c**) Flow cytometric analysis of OT-1 cells after incubation with different concentrations of temoporfin. (**d**) Cell viability of OT-1 cells after incubation with temoporfin. (**e**) Flow cytometric analysis of OT-1 cells after incubation with temoporfin at 4 or 37 °C. (**f**) GMFI of OT-1 cells after incubation with temoporfin at 4 or 37 °C. (**g**) Fluorescence images of PS-OT-1 cells or unloaded OT-1 cells. The asterisk in figures indicate a statistical significance with *p* value of <0.001 ***.

The optimal condition to load the photosensitizer into OT-1 cells was investigated via fluorescence imaging and flow cytometry. After 2 h of incubating the OT-1 cells with different concentrations of temoporfin, ranging between 12.5 and 100 μg/mL, at 37 °C, the cellular uptake of temoporfin was observed. The fluorescence images showed that the loading amount of temoporfin in the OT-1 cells was significantly increased in a concentration-dependent manner, wherein bright red colors of temoporfin were clearly observed in the cytoplasm compartment of the OT-1 cells (Figure 2a). The successful loading of temoporfin (25 μg/mL) into the OT-1 cells was also confirmed through the brown staining of the temoporfin-loaded OT-1 cell (PS-OT-1 cells) sediment compared to that of the unloaded OT-1 cells (Figure 2b). The flow cytometric analysis of the PS-OT-1 cells also revealed elevated fluorescence intensities of temoporfin in the OT-1 cells corresponding to the increased incubation concentrations of temoporfin, ranging between 12.5 and 100 μg/mL (Figure 2c). These results suggest that the loading amount of temoporfin within OT-1 cells is strongly correlated with the loading concentration of temoporfin. However, loading with a concentration of 50 and up to 100 μg/mL of temoporfin induced significant dark cytotoxicity in the OT-1 cells, wherein the cell viability was decreased to levels of less than 20% compared to the non-treated OT-1 cells; furthermore, the toxic effect of temoporfin on the OT-1 cells was worse when the incubation time was increased from 24 to 48 h (Figure 2d). These results clearly demonstrate that a high concentration of temoporfin with a long incubation time can potentially lead to the dysfunction of T cells as a living delivery vesicle. Thus, further studies were performed at an optimized concentration of 25 μg/mL temoporfin and the incubation time was also fixed to 2 h. We also assessed the influence of the incubation temperature on the temoporfin loading efficiency. When OT-I cells were incubated with temoporfin (25 μg/mL) at 4 °C or 37 °C, loading at 37 °C clearly resulted in a much greater fluorescence intensity of the OT-1 cell population, as demonstrated through the flow cytometry (Figure 2e). Quantitatively, a comparison of the corresponding normalized geometric means of fluorescence intensities (GMFI) showed that the GMFI of the T cell population incubated with temoporfin at 37 °C is about 33 times greater than the GMFI of the population loaded at 4 °C (Figure 2f). These results also indicate that the loading mechanism of temoporfin into T cells is probably an energy-dependent endocytic process [21]. At the optimized loading conditions for PS-OT-1 cells, which were prepared through incubation with 25 μg/mL of temoporfin at 37 °C for 2 h, the internalized PS was quantitatively determined through lysing the PS-OT-1 cells and the subsequent analysis of such cell lysates via UV/Vis-spectroscopy, wherein the cell lysates did not affect the UV absorption of temoporfin (Figure S2). As shown in Figure S3, it was confirmed that, on average, 0.060 ± 0.006 pg of temoporfin is taken up by a single T cell. The fluorescence images revealed that, in contrast to the unloaded OT-1 cells, the PS-OT-1 cells showed intense fluorescence signals due to the loaded temoporfin located in the cytoplasm (Figure 2g). Although the signals of the cell membrane (anti-CD8a mAb stained; green color) and nuclei (DAPI stained; blue color) are much stronger than the fluorescence emitted by temoporfin (red color), taken together, temoporfin was sufficiently loaded into the cytosol of the OT-1 cells through an energy-dependent endocytosis mechanism.

### 3.2. In Vitro Combinational Effect of PDT and ACT by PS-OT-1 Cells

**Figure 3:**
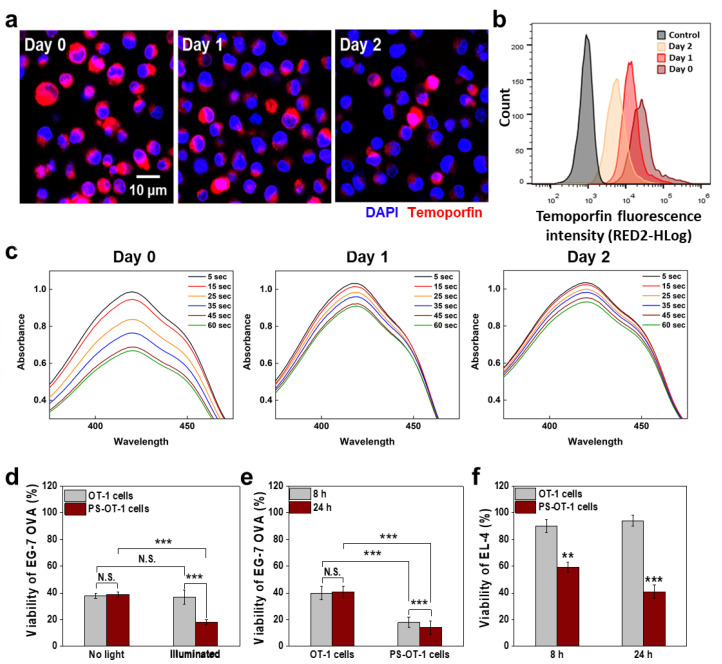
In vitro combinational antitumor effect of PDT and ACT by PS-OT-1 cells. (**a**) Fluorescence images of PS-OT-1 cells after 0, 1 or 2 days of incubation. (**b**) Flow cytometric analysis of PS-OT-1 cells after 0, 1 or 2 days of incubation. (**c**) Extracellular ROS generation of PS-OT-1 cells that were incubated in cell culture media at 37 °C for 2 days. After 6 h of incubation of PS-OT-1 cells at day 1, 2, each cell media was isolated and the extracellular ROS was measured using DPBF bleaching assays. (**d**) Cell viability of EG.7-OVA after incubation with PS-OT-1 cells or unloaded OT-1 cells in presence or absence of the light irradiation. (**e**) Cell viability of EG.7-OVA after incubation with PS-OT-1 cells or unloaded OT-1 cells after 8 or 24 h of incubation. (**f**) Cell viability of EL-4 cells after incubation with PS-OT-1 cells or unloaded OT-1 cells. The asterisks in figures indicate a statistical significance with *p* value of <0.01 ** and <0.001 ***. N.S. in figures indicate a statistically not significant.

To evaluate whether the PS-OT-1 cells had enough temoporfin internalized after reaching the targeted tumor site to exert a combinational effect of PDT and ACT, the amount of photosensitizer that remained in the T cells after drug loading was determined. To this end, the drug release profiles of the PS-OT-1 cells, which had been prepared through incubation with 25 μg/mL temoporfin for 2 h, were assessed by measuring the remaining temoporfin in the T cells over time. First, confocal laser scanning microscopy (CLSM) images showed that the fluorescence signals of temoporfin (red color) gradually decreased in a time-dependent manner, owing to the release of the temoporfin from the T cells and, to some extent, due to their proliferation, where the drug content of mother cells was distributed to daughter cells (Figure 3a). These results are clearly supported by the flow cytometric analysis, which shows a reduction in the temoporfin fluorescence intensities of the PS-OT-1 cell populations over time, compared to day 0, after drug loading; 77% and 56% of the temoporfin was left in the PS-OT-1 cells on days 1 and 2, respectively (Figure 3b). This is the result of the relatively slow proliferation time of T cells compared to cancer cells, for instance, it was reported that fluorescent dye loaded into T cells was sustainably retained for four days due to their delayed doubling time. Next, the extracellular ROS generation from the PS-OT-1 cells under light irradiation was evaluated via 1,3-diphenylisobenzofuran (DPBF) bleaching assays. We expect that PS-OT-1 cells can deliver photosensitizer molecules as living vehicles to targeted tumor tissues via the T cells’ homing effect. Subsequently, they can deliver the photosensitizer to tumor cells by releasing the loaded photosensitizer. Therefore, to measure the temoporfin release effect of the PS-OT-1 cells, DPBF was used for the analysis of the extracellular ROS generation when PS was released over incubation times of 6 h and one or two days. As expected, the freshly prepared PS-OT-1 cells produced a large amount of ROS for 6 h in the cell media upon visible light irradiation for 1 min, which was gradually increased in a light-irradiation-time-dependent manner (day 0; Figure 3c). Importantly, the PS-OT-1 cells still generated significant amounts of ROS after incubation periods of one or two days due to the remaining photosensitizer molecules in the T cells. However, the amount of ROS generated by the PS-OT-1 cells at days 1 and 2 was reduced to 80% and 52%, respectively, compared to the amount of ROS produced by the freshly prepared PS-OT-1 cells at day 0. These results indicate that PS-OT-1 cells could release intracellular-loaded temoporfin slowly over a period of two days, resulting in the successful generation of extracellular ROS upon visible light irradiation.

The combinational antitumor effect of PDT and ACT through PS-OT-1 cells was evaluated after incubation with EG.7-OVA murine lymphoma cells, which can be recognized by OT-1 cells in co-cultures. First, we investigated whether a loading of temoporfin inhibits the lytic activity of OT-I cells. To this end, the bioreductive activity of EG.7-OVA murine lymphoma cells was compared after incubation with unloaded or PS-loaded OT-1 cells without light irradiation for 8 h (Figure 3d). Interestingly, the viability of the EG.7-OVA cells was significantly reduced to 38 ± 2% or 37 ± 2% when they were incubated with unloaded or PS-loaded OT-1 cells without light irradiation, respectively. Consequently, these results demonstrate that temoporfin loading does not impair the natural lytic activity of OT-I cells against target cells. More importantly, the co-culture of EG.7-OVA cells with PS-OT-1 cells after visible light irradiation significantly decreased the viability of the target cells to 18% after 8 h of incubation, whereas the viability of the EG.7-OVA cells co-cultured with unloaded OT-1 cells at the same condition was determined to be only 37%, which is consistent with the viability measured in the absence of light irradiation. These results indicate a combinational antitumor effect of T cells’ lytic activity and PDT against target cells compared to ACT alone. When the incubation time of the co-culture was increased from 8 to 24 h, the differences in the bioreductive activity were more significant, wherein the viability of the EG.7-OVA cells was greatly decreased to 15% after being co-cultured with PS-OT-1 cells compared to the unloaded OT-1 cells (40%; Figure 3e). As a control, EL-4 cells, which are not specific targets for OT-I cells, were also tested; the bioreductive activity of those cancer cells was almost not affected, except by some non-specific lysis following the treatment with T cells. Therefore, we can confirm that PS-OT-1 cells induce significant cytotoxicity in the neighboring non-target cancer cells that are not directly recognized by T cells through promoting ROS via temoporfin-mediated PDT. As expected, the co-culture with unloaded OT-1 cells after visible light irradiation did not significantly decrease the viability of the EL-4 cells after 8 or 24 h of incubation (Figure 3f). In contrast, the viability of the EL-4 cells was significantly decreased after being co-cultured with PS-OT-1 cells for 8 or 24 h after visible light irradiation, to about 52% and 30%, respectively. These results clearly demonstrate that the PS-OT-1 cells induced cytotoxicity against non-specific cancer cells only through PDT following visible light irradiation. From these results, we can expect that PS-OT-1 cells would efficiently eradicate the tumor cells that are directly attacked by T cells, as well as the neighboring cancer cells in the tumor tissues, through the combinational antitumor effect of PDT and ACT.

### 3.3. In Vivo Biodistribution and Antitumor Efficacy of PS-OT-1 Cells in EG.7-OVA Tumor-bearing Mice

**Figure 4:**
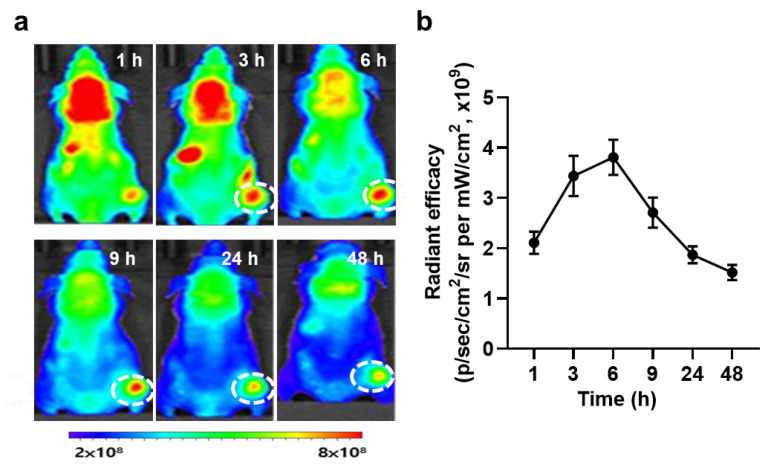
Biodistribution of PS-OT-1 cells. (**a**) In vivo fluorescence images of a tumor treated with PS-OT-1 cells. (**b**) Quantitative analysis of fluorescence intensity in the tumor region.

The in vivo biodistribution of PS-OT-1 cells was assessed in EG.7-OVA tumor-bearing mice. Briefly, the mice were prepared through the subcutaneous inoculation of 4 × 10^6^ EG.7-OVA cells; then, 5 × 10^6^ unloaded or PS-loaded OT-1 cells were intravenously injected when the tumor volumes were approximately 100 mm^3^. Non-invasive near-infrared fluorescence (NIRF) imaging showed a significant tumor accumulation of PS-OT-1 cells (red color) after the injection (Figure 4a). In addition, the influx of PS-OT-1 cells into the tumor tissue (white dotted circle) gradually increased for at least 6 h after the injection, and those cells were sustainably retained in the tumor tissues for at least 48 h post-injection (Figure 4b). This rapid accumulation of PS-OT-1 cells is highly suitable to elicit combinational PDT and ACT because the temoporfin loaded in the PS-OT-1 cells is released over time after administration in vivo.

**Figure 5:**
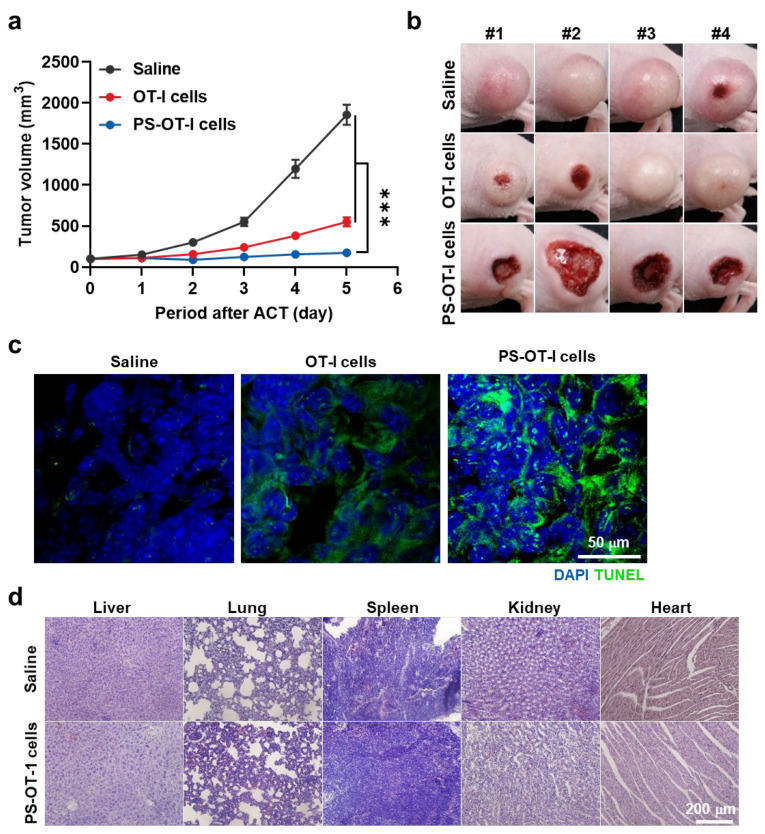
Antitumor efficacy of PS-OT-1 cells. (**a**) Tumor growth of mice treated with saline, unloaded OT-1 cells or PS-OT-1 cells. (**b**) Optical images of mice treated with saline, unloaded OT-1 cells or PS-OT-1 cells. (**c**) Tumor tissues stained with TUNEL of mice treated with saline, unloaded OT-1 cells or PS-OT-1 cells. (**d**) Normal organs stained with H&E. The asterisk in figures indicate a statistical significance with *p* value of <0.001 ***.

The antitumor efficacy of combinational PDT and ACT through PS-OT-1 cells was further assessed in EG.7-OVA tumor-bearing mice after treatment with the same protocol as described above. Next, 6 h after injection, tumor tissues were locally irradiated with 200 J/cm^2^ for 10 min via a laser emitting light with a wavelength of 655 nm, as the PS-OT-1 cells showed the highest tumor accumulation at this time point. Importantly, the superior antitumor efficacy seen in the mice treated with PS-OT-1 cells and irradiation was sustainably observed five days after treatment, wherein the growth of the tumor volumes (173.4 ± 11.5 mm^3^) was significantly delayed compared to the mice treated with saline (1855.61 ± 123.51 mm^3^) or unloaded OT-1 cells (550.61 ± 50.61 mm^3^) (Figure 5a). Optical images of the tumors revealed that the mice in the saline group showed rapid tumor growth, whereas the tumor growth was inhibited in the mice treated with OT-1 cells and irradiation. However, the temoporfin-loaded OT-1 cells in combination with irradiation had the strongest inhibitory effect on tumor growth (Figure 5b), indicating the potent antitumor efficacy of combinational PDT and ACT. Finally, tumor tissues stained with TUNEL showed extensive apoptosis in the laser irradiated PS-OT-1 cell-treated mice compared to the mice treated with saline or unloaded OT-1 cells on day five of treatment (Figure 5c). These in vivo results clearly demonstrate the potent antitumor efficacy of combinational PDT and ACT through PS-OT-1 cells compared to ACT alone. Finally, the PS-OT-1 cell-treated and laser-irradiated mice did not show any toxicity in the liver, lung and spleen, wherein the healthy tissues showed a similar morphology to the saline-treated mice five days post-treatment (Figure 5d). Collectively, these in vivo results clearly demonstrate the potent antitumor efficacy of combinational PDT and ACT through PS-OT-1 cells compared to PDT alone or ACT alone.

This approach is a facile and generalizable strategy to induce a significant apoptotic cell death of target tumor cells. In addition, these results suggest that photosensitizer-loaded T cells are a promising strategy for effective cancer immunotherapy.

## 4. Conclusions

In this study, we loaded photosensitizer molecules into cytotoxic T lymphocytes to improve the antitumor efficacy of ACT via a combinational PDT and cancer immunotherapy. First, the optimal methodology to prepare photosensitizer-loaded cytotoxic T lymphocytes was established. The loading amount of the photosensitizer was highly dependent on both the concentration and temperature. In addition, we also determined the exact loading amount of the photosensitizer internalized by cytotoxic T lymphocytes for this optimized condition. Through in vitro experiments, the drug release profiles and extracellular ROS generation after visible light irradiation of the photosensitizer-loaded cytotoxic T lymphocytes were assessed. Importantly, the combination of PDT and ACT using PS-OT-1 cells killed more target cancer cells. Finally, the antitumor efficacy of PS-OT-1 cells was assessed in EG.7-OVA tumor-bearing mice after intravenous injection, which significantly delayed tumor growth. Overall, these results suggest that combinational PDT and ACT through photosensitizer-loaded cytotoxic T lymphocytes provides a new route for effective cancer immunotherapy.

## Data Availability

All relevant data are available with the article and its Supplementary Information files, or available to the corresponding authors upon reasonable requests.

## Supplementary Materials

The following supporting information can be downloaded at: https://www.mdpi.com/article/10.3390/pharmaceutics15041295/s1. Figure S1. The CD8 expression of OT-1 cells, as confirmed by Flow cytometer. Figure S2. Quantitative analysis of loaded temoporfin in the OT-1 cells. Figure S3. Fluorescence intensity profile of PS-OT-1 cells.
