# Supplementary material for: Adoptive Transfer of Photosensitizer-Loaded Cytotoxic T Cells for Combinational Photodynamic Therapy and Cancer Immuno-Therapy
Source: Pharmaceutics. 2023 Apr 20;15(4):1295. doi: 10.3390/pharmaceutics15041295 (PMC10143374; doi:10.3390/pharmaceutics15041295)
Supplement: Supplementary file 1 [file pharmaceutics-15-01295-s001.zip › pharmaceutics-2325997-supplementary.pdf]

Supplementary Information for

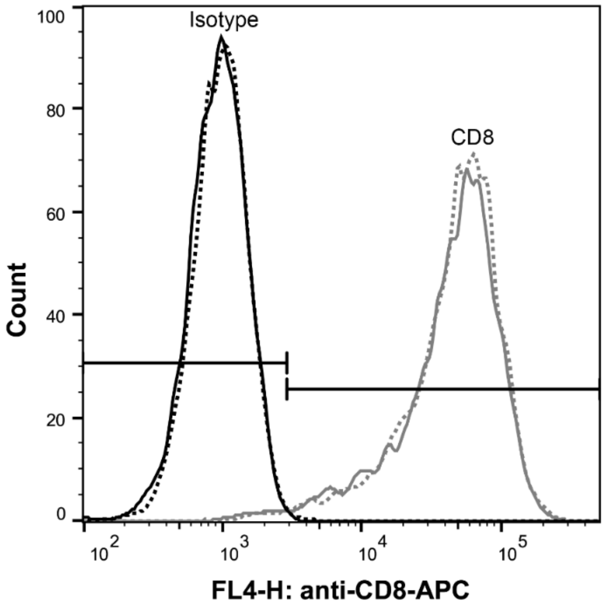

Figure S1. The CD8 expression of OT-1 cells, as confirmed by Flow cytometer.

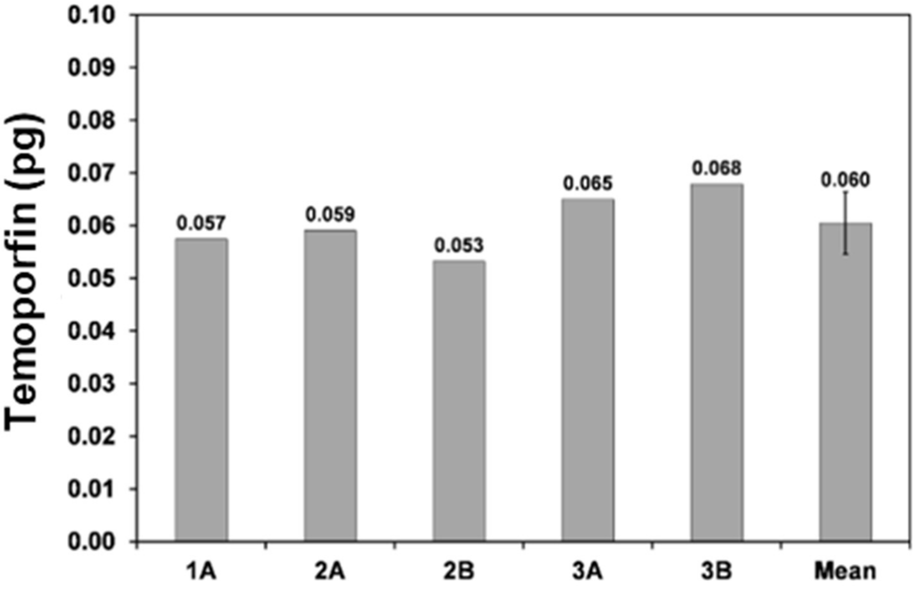

Figure S2. Quantitative analysis of loaded temoporfin in the OT-1 cells.

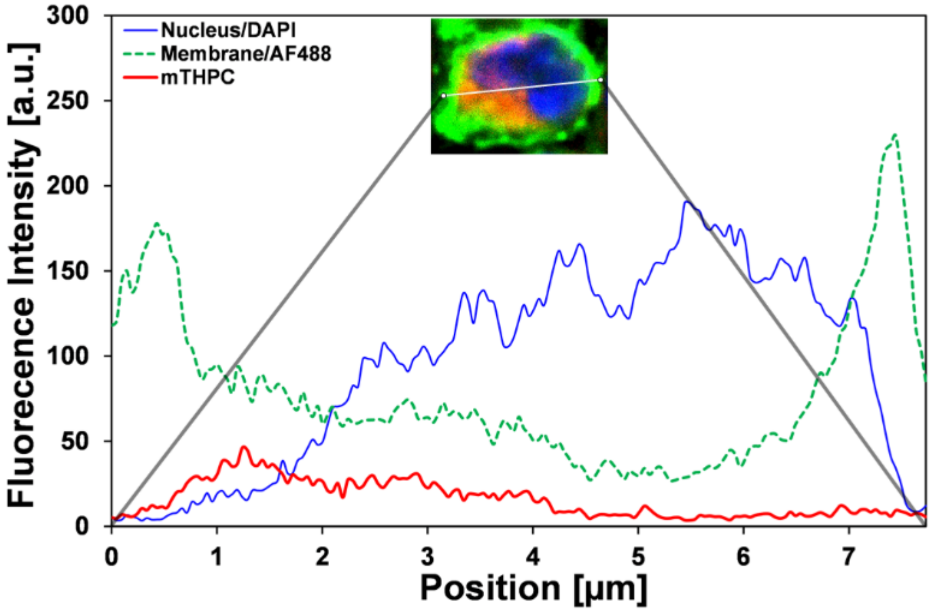

Figure S3. Fluorescence intensity profile of PS-OT-1 cells.
